# Complementary Role of Fibroblast Growth Factor 21 and Cytokeratin 18 in Monitoring the Different Stages of Nonalcoholic Fatty Liver Disease

**DOI:** 10.1038/s41598-017-05257-5

**Published:** 2017-07-11

**Authors:** Guangyu Wu, Huating Li, Qichen Fang, Jing Zhang, Mingliang Zhang, Lei Zhang, Liang Wu, Xuhong Hou, Junxi Lu, Yuqian Bao, Weiping Jia

**Affiliations:** 1 Department of Endocrinology and Metabolism, Shanghai Jiao Tong University Affiliated Sixth People’s Hospital; Shanghai Key Laboratory of Diabetes Mellitus, Shanghai Clinical Center of Diabetes, Shanghai, 200233 China; 2 Department of Medicine, Shanghai Jiao Tong University School of Medicine, Shanghai, 200025 China

## Abstract

Fibroblast growth factor 21 (FGF21) and cytokeratin 18 (CK18) were previously reported to be elevated in nonalcoholic fatty liver disease (NAFLD). We aim to analyze the differential roles of FGF21, cell apoptosis marker CK18 fragment M30 and total cell death marker CK18 M65ED in monitoring the different stages of NAFLD spectrum in a population-based prospective cohort comprising 808 Chinese subjects. Predictive performances for monitoring the different stages of NAFLD were assessed by logistic regression and receiver-operating characteristic (ROC) curves. We found baseline FGF21 but not CK18 level was an independent predictor for the development of simple steatosis. NAFLD patients who had remission during follow-up had significantly lower baseline M30 levels than those who sustained NAFLD (84.74U/L [53.26–135.79] *vs*. 118.47U/L [87.16–188.89], P = 0.012). M65ED was independently predictive of progressing to suspected non-alcoholic steatohepatitis (NASH) in NAFLD patients. These results suggest that FGF21 can be used for early identification of hepatic steatosis. On the other hand, CK18 including M30 and M65ED, are predictive of the prognosis of NAFLD patients. FGF21 and CK18 might play differential roles and have complementary value in non-invasive identification and monitoring the outcome of NAFLD patients.

## Introduction

Non-alcoholic fatty liver disease (NAFLD) is defined by the presence of liver fat accumulation exceeding 5% of hepatocytes, in the absence of significant alcohol intake, viral infection, or any other specific etiology of liver disease^1^. NAFLD is divided into simple steatosis and nonalcoholic steatohepatitis (NASH) which is distinguished from the former by additional presence of hepatocellular injury with or without fibrosis^2^. The majority of patients with simple steatosis are stable in term of liver histology over time, while NASH is associated with progressive liver disease^3^. The gold standard for diagnosing and differentiating simple steatosis and NASH is liver biopsy, but it is invasive and limited by sampling error^4^. In clinical practice, ultrasonography is the most commonly used imaging technique for diagnosing NAFLD, while it shows low accuracy in diagnosing NAFLD in obese patients. The sensitivity also decreases dramatically for mild steatosis^5^. Magnetic resonance spectrophy (MRS) is more sensitive and accurate but it is expensive and not broadly available^6^. Neither ultrasonography nor MRS can differentiate between patients with simple steatosis and NASH. In an attempt to overcome biopsy and to monitor NAFLD patients leading to better intervention decisions, seeking potential novel biomarkers based on current knowledge of the pathophysiology of NAFLD are yet far from being accomplished.

Previous studies reported FGF21 and CK18 fragment are potential NAFLD biomarkers^6, 7^. Fibroblast growth factor 21 (FGF21) is predominantly released from hepatocytes and in a lesser extent from adipocytes and other tissues^8^. FGF21 binds to fibroblast growth factor receptor and co-receptor beta-klotho and exerts its hormone-like metabolic effects^8^. FGF21 knockout mice are refractory to the beneficial insulin-sensitizing effects of the peroxisome proliferator-activated receptor (PPAR)γ agonist rosiglitazone^9^. FGF21 deficiency also exacerbated accumulation of triglycerides (TG), impaired activation of fatty acids and oxidation in the liver and increased inflammation and fibrosis^10^. Despite the beneficial effect of FGF21 found in animal studies, circulating FGF21 levels in human are elevated in obesity, metabolic syndrome, type 2 diabetes and coronary artery disease^11^. We previously found FGF21 level was increased in NAFLD patients and was an independent predictor of NAFLD^7, 12^. The elevation of circulating FGF21 levels in over-nutrition may indicate the presence of compensatory responses of FGF21 to the underlying metabolic stress^11^. Cytokeratin 18 (CK18) is a major intermediate filament protein of hepatocytes^13^. CK18 is cleaved by caspases and released to the circulation during hepatocyte apoptosis, which is a characteristic feature of liver injury and disease progression in NAFLD^13, 14^. The serum level of cleaved CK18 fragment is representative of the degree of hepatocyte apoptosis and can be measured by M30 level, which is an epitope generated during CK18 cleavage. In contrast, M65 antigen, existing on both cleaved and uncleaved CK18 protein, is used as a marker for total death of hepatocytes, including both apoptosis and necrosis^15^. M65 EpiDeath (M65ED) ELISA uses inverse capture antibody and detection antibody compared to M65 ELISA and further improves binding specificity^15^. Recent clinical studies demonstrated that M30 and M65ED are potentially useful to diagnose fatty liver, NASH and fibrosis^13, 15^. A two-step approach using M30 and FGF21 was demonstrated to further improve the accuracy in diagnosing NASH^16^.

Based on the above mentioned research, it seems that FGF21 and CK18 are involved in the different stages in view of the pathophysiology of NAFLD. However, no longitudinal epidemiological study focused on the differential roles of FGF21 and CK18 in NAFLD spectrum. In this prospective study, we aimed at assessing FGF21, CK18 M30 and M65ED levels during different stages in the spectrum of NAFLD and evaluating their respective role in early identification and predicting the prognosis of NAFLD patients to investigate their clinical significance.

## Results

### Clinical Characteristics of subjects and serum FGF21, M30 and M65ED levels at baseline

As described in our previous study^7^, 660 subjects from baseline survey (257 men and 403 women; aged 49.15 ± 12.60 years) were included in this follow-up reassessment after a mean duration of 3.1 ± 0.1 years, 148 subjects were not followed up due to emigration, refusal or death. Subjects enrolled in the follow-up study were compared to those who were not included and no difference was observed between groups in demographic, anthropometric and biochemical indexes. Ninety-five subjects were not included in the analysis according to exclusive criteria such as acute or chronic virus hepatitis and excessive alcohol consumption. In subjects without NAFLD at baseline, 70 subjects developed simple steatosis, 9 subjects developed suspected NASH and 363 subjects did not develop NAFLD, no one was treated with medications before follow-up assessment. In 123 subjects with NAFLD at baseline, 93 subjects sustained NAFLD and 30 subjects had remission of NAFLD at follow-up assessment. Among subjects who sustained NAFLD, 24 subjects had suspected NASH at follow-up reassessment. A subgroup of eight subjects from suspected NASH in 2011 was followed up for another 5 years (Supplementary Fig. S1).

The baseline clinical and laboratory characteristics of subjects are described in Table 1. After adjustment for age, gender and BMI, patients with NAFLD had higher waist circumference, fat percentage, blood pressure, liver enzymes, TG, fasting plasma glucose (FPG), 2-hour plasma glucose (2hPG), hemoglobin A1c (HbA_1c_), homeostasis model assessment (HOMA)-IR than those of non-NAFLD subjects (all P < 0.01). Serum adiponectin levels and HOMA-beta in NAFLD subjects were significantly lower than those in non-NAFLD group after adjustment for age, gender and body mass index (BMI) (both P < 0.05). We next investigated the cross-sectional relationship between NAFLD and serum FGF21, M30 and M65ED levels. Besides FGF21 level, circulating levels of M30 and M65ED in patients with NAFLD were also significantly higher than those in non-NAFLD subjects after adjustment for age, gender and BMI (all P < 0.01).

**Table 1:** Baseline characteristics of subjects with non-NAFLD and NAFLD.

Baseline variables	Non-NAFLD (n = 442)	NAFLD (n = 123)	*P* value non-NAFLD *vs*. NAFLD	
			Adjusted for age and gender	Adjusted for age, gender and BMI
M/F	159/283	59/64	—	—
Age (years)	45.74 ± 13.13	48.50 ± 10.95	—	—
BMI (kg/m^2^)	22.57 ± 2.87	27.33 ± 2.50	<0.001	—
Waist circumference (cm)	73.39 ± 7.96	86.78 ± 6.71	<0.001	<0.001
Fat percentage (%)	25.70 ± 6.68	33.42 ± 7.22	<0.001	<0.001
SBP (mmHg)	122.91 ± 16.82	135.78 ± 15.45	<0.001	0.002
DBP (mmHg)	80.38 ± 9.53	88.49 ± 9.48	<0.001	0.002
ALT (IU/L)^§^	14.00 (11.00–19.00)	25.50 (15.00–42.00)	<0.001	<0.001
AST (IU/L)^§^	19.00 (16.00–23.00)	23.00 (19.00–28.25)	<0.001	<0.001
GGT (IU/L)^§^	16.00 (13.00–23.00)	28.50 (19.75–48.25)	<0.001	<0.001
TC (mmol/L)	4.65 ± 1.01	5.00 ± 0.90	0.003	0.087
TG (mmol/L)^§^	1.09 (0.76–1.58)	2.16 (1.43–3.33)	<0.001	<0.001
HDL-C (mmol/L)	1.39 ± 0.29	1.19 ± 0.26	<0.001	0.008
LDL-C (mmol/L)	2.85 ± 0.84	3.00 ± 0.81	0.179	0.745
FPG (mmol/L)	5.17 ± 1.01	5.83 ± 1.61	<0.001	<0.001
2hPG (mmol/L)	6.20 ± 2.33	8.30 ± 3.75	<0.001	<0.001
HbA1c (%)	5.63 ± 0.77	6.01 ± 0.91	<0.001	0.003
HOMA-beta^§^	70.16 (46.70–99.38)	64.49 (42.80–90.46)	0.254	0.021
HOMA-IR^§^	1.19 (0.82–1.64)	1.51 (1.05–2.10)	<0.001	0.254
Adiponectin (μg/ml)^§^	8.77 (6.20–13.08)	5.26 (3.02–7.13)	<0.001	<0.001
FGF21 (pg/ml)^§^	222.44 (136.06–351.47)	357.50 (230.67–592.66)	<0.001	<0.001
CK18 M30 (U/L)^§^	86.29 (57.92–136.20)	113.78 (81.53–172.17)	<0.001	0.001
CK18 M65ED (U/L)^§^	150.70 (103.90–239.90)	266.35 (154.78–437.13)	<0.001	<0.001

Data are means ± SD or median (interquartile range). ^§^Ln transformed before analysis.

The Pearson’s and partial correlations among baseline FGF21, M30 and M65ED levels and clinical parameters in total cohort were shown in Supplementary Table S1. Both FGF21 and M65ED levels were positively associated with age and BMI (P < 0.001). M30 was significantly correlated with BMI (P = 0.032). After adjustment for age and BMI, M30 level was found to be positively associated with biochemical indicators of liver injury including alanine aminotransferase (ALT), aspartate transaminase (AST) and γ-glutamyltransferase (GGT) as well as total cholesterol (TC), low density lipoprotein-cholesterol (LDL-C) and 2hPG (all P < 0.05). Serum M65ED level was positively related to waist circumference, systolic blood pressure (SBP), diastolic blood pressure (DBP), ALT, AST, GGT, TC, TG, FPG, 2hPG and HbA_1c_ (all P < 0.01). Negative association was found between M65ED with high density lipoprotein-cholesterol (HDL-C) and adiponectin (P < 0.05).

### Baseline FGF21 but not M30 or M65ED level can predict the onset of simple steatosis

**Figure 1:**
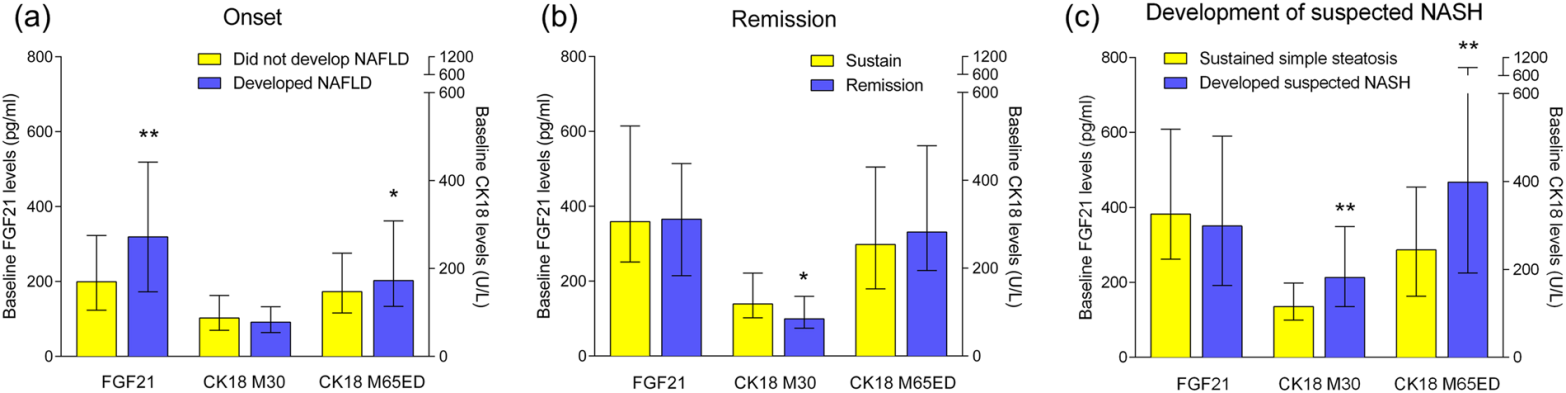
Comparison of FGF21, M30 and M65ED levels at baseline in different groups. (**a**) Baseline FGF21, M30, M65ED levels in subjects who developed simple steatosis (n = 70) and who did not (n = 363). (**b**) Baseline FGF21, M30, M65ED levels in subjects who sustained NAFLD (n = 93) and who had remission during follow-up (n = 30). (**c**) Baseline FGF21, M30 and M65ED levels in subjects who sustained simple steatosis (n = 69) and subjects who developed suspected NASH (n = 24). Data represent median (interquartile range). *P < 0.05, **P < 0.01, ***P < 0.001.

In subjects without NAFLD at baseline, characteristics of 70 subjects who developed simple steatosis during 3-year follow-up and 363 subjects who did not develop simple steatosis were described in Supplementary Table S2. Baseline FGF21 was increased in subjects who developed simple steatosis during follow-up (309.79 pg/ml [169.11–506.43]) than those who did not (199.10 pg/ml [123.56–322.80], P < 0.001, Fig. 1a, Supplementary Table S2). However, no significant difference was found in M30 levels between the two groups. M65ED levels were slightly elevated in subjects who developed simple steatosis (172.65U/L [112.98–301.05] *vs*. 147.20U/L [98.90–234.90], P = 0.049, Fig. 1a, Supplementary Table S2).

Independent predictors for simple steatosis were identified using multiple logistic regression. Baseline clinical parameters (age, gender, BMI, waist circumference, SBP and DBP, ALT, AST, GGT, TC, TG, HDL-C, LDL-C, FPG and 2hPG), HOMA-beta, HOMA-IR and adiponectin with FGF21, M30 and M65ED were included respectively in the models (Table 2). Baseline FGF21 but not M65ED level is an independent predictor for simple steatosis. These results suggested that FGF21 has obvious advantage in early diagnosis of hepatic steatosis compared to CK18 and CK18 fragment.

**Table 2:** Baseline parameters predictive of the onset of simple steatosis at 3 years, examined using multiple logistic regression.

Baseline variables	Model 1	Model 2	Model 3	Model 4	Model 5
Age (years)	1.028 (0.990–1.068)	1.025 (0.987–1.065)	1.034 (0.993–1.076)	1.024 (0.990–1.058)	1.020 (0.985–1.057)
Gender	2.154 (0.907–5.116)	1.985 (0.817–4.819)	2.210 (0.907–5.388)	3.015 (1.254–7.250)*	2.454 (1.048–5.744)*
BMI (kg/m^2^)	1.345 (1.079–1.677)*	1.324 (1.058–1.656)*	1.394 (1.109–1.754)*	1.304 (1.056–1.609)*	1.314 (1.069–1.614)*
Waist circumference (cm)	1.037 (0.956–1.124)	1.042 (0.960–1.131)	1.025 (0.944–1.114)	1.060 (0.982–1.144)	1.060 (0.983–1.142)
SBP (mmHg)	0.968 (0.936–1.002)	0.966 (0.933–1.001)	0.969 (0.937–1.003)	0.975 (0.944–1.007)	0.977 (0.947–1.009)
DBP (mmHg)	1.068 (1.009–1.130)*	1.076 (1.015–1.141)*	1.069 (1.010––.131)*	1.057 (1.001–1.117)*	1.054 (0.999–1.113)
ALT (IU/L)^§^	1.787 (0.495–6.450)	1.854 (0.498–6.905)	1.962 (0.529–7.278)	2.053 (0.584–7.215)	1.405 (0.413–4.774)
AST (IU/L)^§^	0.613 (0.075–5.029)	0.707 (0.082–6.081)	0.735 (0.088–6.121)	0.569 (0.080–4.053)	0.628 (0.083–4.767)
GGT (IU/L)^§^	0.977 (0.432–2.210)	0.847 (0.359–1.996)	0.821 (0.353–1.912)	1.150 (0.522–2.535)	1.024 (0.465–2.257)
TC (mmol/L)	1.086 (0.490–2.408)	1.173 (0.514–2.675)	1.153 (0.520–2.558)	1.207 (0.540–2.697)	1.191 (0.547–2.593)
TG (mmol/L)^§^	1.112 (0.411–3.008)	0.960 (0.342–2.696)	1.007 (0.366–2.772)	0.925 (0.343–2.494)	1.192 (0.455–3.119)
HDL-C (mmol/L)	0.696 (0.120–4.034)	0.566 (0.092–3.492)	0.799 (0.136–4.707)	0.434 (0.076–2.474)	0.631 (0.117–3.398)
LDL-C (mmol/L)	0.882 (0.407–1.909)	0.814 (0.365–1.814)	0.833 (0.385–1.804)	0.833 (0.378–1.834)	0.870 (0.408–1.852)
FPG (mmol/L)	0.811 (0.524–1.253)	1.075 (0.517–2.237)	0.790 (0.505–1.236)	0.735 (0.484–1.117)	0.746 (0.496–1.123)
2hPG (mmol/L)	1.122 (0.963–1.308)	1.117 (0.962–1.296)	1.114 (0.954–1.301)	1.165 (1.007–1.348)*	1.151 (0.998–1.328)
HOMA-beta^§^	/	1.712 (0.482–6.085)	/	/	/
HOMA-IR^§^	/	0.807 (0.203–3.204)	/	/	/
Adiponectin^§^	/	/	0.622 (0.319–1.212)	/	/
FGF21 (pg/ml)^§^	1.814 (1.063–3.096)*	1.861 (1.078–3.213)*	1.881 (1.096–3.228)*	/	/
CK18 M30 (U/L)^§^	/	/	/	0.665 (0.370–1.197)	/
CK18 M65ED (U/L)^§^	/	/	/	/	1.211 (0.689–2.128)

Data are odds ratio (OR) (95%CI).

^§^Ln transformed before analysis. *P < 0.05.

Model 1. Included baseline risk factors (age, gender, BMI, waist circumference, SBP, DBP, ALT, AST, GGT, TG, TG, HDL-C, LDL-C, FPG and 2hPG), and FGF21.

Model 2 Included baseline risk factors, HOMA-beta, HOMA-IR, and FGF21.

Model 3 Included baseline risk factors, adiponectin, and FGF21.

Model 4 Included baseline risk factors and CK18 M30.

Model 5 Included baseline risk factors and CK18 M65ED.

### Baseline M30 level is an independent predictor of NAFLD remission

In NAFLD patients, hepatocyte apoptosis was reported to be substantially increased in patients with NASH and correlated with the prognosis of the disease^14^. Therefore, we investigated if levels of cell death biomarkers were correlated with the remission of NAFLD. Baseline characteristics of patients who had remission of NAFLD and who sustained NAFLD were presented in Table 3. Subjects who had remission of NAFLD had significantly lower M30 levels at baseline (84.74 U/L [53.26–135.79]) than subjects who sustained NAFLD (118.47 U/L [87.16–188.89], P = 0.012). However, these two groups had similar baseline serum FGF21 and M65ED levels (Fig. 1b). These two groups also had similar baseline ALT levels, as well as AST, GGT, TC, TG and HDL-C levels. Subjects had remission of NAFLD also had significantly lower BMI and waist circumference, along with a non-significant trend of lower LDL-C and FPG than those of subjects who sustained NAFLD. The proportion of subjects who had regular exercise was significantly higher in subjects who had remission of NAFLD than those who sustained NAFLD during follow-up (Table 3).

**Table 3:** Baseline clinical parameters of 123 subjects who had remission of NAFLD or sustained NAFLD after 3-year follow-up.

Baseline variables	NAFLD state at 3 years		*P* value
	Remission of NAFLD (n = 30)	Sustained NAFLD (n = 93)	
M/F	14/16	45/48	0.912
Age (years)	50.60 ± 10.13	47.83 ± 11.23	0.232
BMI (kg/m^2^)	26.52 ± 1.81	27.59 ± 2.66	0.016
Waist circumference (cm)	84.00 ± 5.51	87.68 ± 6.88	0.009
Fat percentage (%)	32.79 ± 9.09	33.72 ± 6.52	0.544
SBP (mmHg)	134.33 ± 14.98	136.32 ± 15.72	0.545
DBP (mmHg)	88.87 ± 11.36	88.37 ± 8.90	0.807
ALT (IU/L)^§^	24.00 (14.00–37.00)	26.00 (15.00–46.00)	0.321
AST (IU/L)^§^	23.00 (19.75–26.50)	23.00 (18.00–29.00)	0.969
GGT (IU/L)^§^	28.00 (17.75–52.75)	29.00 (20.00–48.00)	0.969
TC (mmol/L)	4.80 ± 0.96	5.08 ± 0.86	0.141
TG (mmol/L)^§^	1.63 (1.28–4.23)	2.23 (1.50–3.31)	0.885
HDL-C (mmol/L)	1.17 ± 0.32	1.19 ± 0.24	0.762
LDL-C (mmol/L)	2.77 ± 0.74	3.09 ± 0.81	0.054
FPG (mmol/L)	5.44 ± 0.95	5.93 ± 1.74	0.052
2hPG (mmol/L)	7.67 ± 3.56	8.43 ± 3.78	0.340
HbA1c (%)	5.86 ± 0.94	6.03 ± 0.87	0.372
HOMA-beta^§^	62.71 (42.12–93.85)	67.80 (42.89–91.08)	0.755
HOMA-IR^§^	1.39 (0.94–2.27)	1.59 (1.17–2.10)	0.151
Regular exercise (%) during follow-up	46.67	21.51	0.008
Low calorie diet (%) during follow-up	30.00	26.88	0.764
Adiponectin (μg/ml)^§^	6.06 (3.40–8.70)	5.19 (2.98–6.64)	0.161
FGF21 (pg/ml)^§^	365.61 (214.59–514.09)	358.86 (251.03–614.59)	0.256
CK18 M30 (U/L)^§^	84.74 (63.26–135.79)	118.47 (87.16–188.89)	0.012
CK18 M65ED (U/L)^§^	282.10 (194.80–478.85)	254.00 (152.90–430.00)	0.728

Data are means ± SD or median (interquartile range). ^§^Ln transformed before analysis.

Using univariate logistic regression analysis, the odds ratio (OR) for a 1-unit increase in ln M30 in predicting remission of NAFLD during 3-year follow-up was 0.346 (95%CI 0.147–0.814; P = 0.015, Supplementary Table S3). Baseline parameters that were different between subjects who had and who did not have remission of NAFLD were included in multivariate logistic regression. When BMI, waist circumference, LDL-C, FPG and M30 were included in the regression model, only waist circumference (OR: 0.899 [95%CI 0.809–0.999]; P = 0.047) and lower M30 level (OR: 0.364 [95%CI 0.141–0.936]; P = 0.036) were independent predictors of NAFLD remission. When regular exercise and low calorie diet were included in regression model along with M30, both regular exercise (OR: 2.845 [95%CI 1.097–7.380]; P = 0.032) and lower baseline M30 level (OR: 0.356 [95%CI 0.147–0.859]; P = 0.022) were independent predictors of NAFLD remission (Supplementary Table S3). The result suggested that the remission of NAFLD during follow-up was independently associated with the level of hepatocyte apoptosis at baseline, which is reflected by circulating M30 levels, regardless of ALT levels at baseline and the benefit of lifestyle change afterwards.

### Baseline M30 and M65ED levels are associated with suspected NASH at follow-up

Although NASH cannot be excluded from NAFLD patients with normal ALT levels, it is suggested that patients with NAFLD and increased ALT levels are at higher risk of suffering NASH^6^. Therefore, we investigated the levels of baseline FGF21 and CK18 in “sustained simple steatosis” group and “developed suspected NASH” group. Suspected NASH was defined as NAFLD patients with ALT level ≥40 U/L^17, 18^. Baseline characteristics of subjects who sustained simple steatosis and who developed to suspected NASH during follow-up were presented in Table 4. It was demonstrated that younger and male subjects were prone to progress to suspected NASH at follow up. These two groups had similar BMI, waist circumference, TC, TG, HDL-C, LDL-C, FPG, 2hPG, HOMA values and adiponectin at baseline. Subjects who had suspected NASH at follow-up reassessment had increased baseline AST and GGT levels compared to sustained simple steatosis. Notably, baseline M30 and M65ED levels were significantly higher in subjects who had suspected NASH at follow-up reassessment than those who sustained simple steatosis, while FGF21 level were similar in these two groups (Fig. 1c).

**Table 4:** Baseline parameters in patients who sustained simple steatosis (n = 69) and patients who developed suspected NASH (n = 24) at follow-up.

Baseline parameters	Sustained simple steatosis (n = 69)	Suspected NASH (n = 24)	*P* values
M/F	28/41	17/7	0.013
Age (years)	50.22 ± 10.34	40.81 ± 11.33	0.001
BMI (kg/m^2^)	27.76 ± 2.55	27.18 ± 3.1	0.392
Waist circumference (cm)	87.72 ± 7.11	87.81 ± 6.5	0.961
Fat percentage (%)	34.31 ± 6.45	31.41 ± 6.67	0.088
SBP (mmHg)	138.81 ± 16.34	129.43 ± 11.07	0.016
DBP (mmHg)	89.10 ± 9.18	86.29 ± 8.19	0.211
AST (IU/L)^§^	20.5 (17.0–27.0)	27.0 (22.5–40.5)	0.003
GGT (IU/L)^§^	26.0 (19.0–38.5)	40.0 (29.5–53.0)	0.012
TC (mmol/L)	5.11 ± 0.8	5.07 ± 1.02	0.823
TG (mmol/L)^§^	2.16 (1.45–3.03)	2.42 (1.85–3.61)	0.222
HDL-C (mmol/L)	1.21 ± 0.25	1.14 ± 0.19	0.220
LDL-C (mmol/L)	3.10 ± 0.76	3.11 ± 0.98	0.945
FPG (mmol/L)	5.86 ± 1.70	5.95 ± 1.66	0.826
2hPG (mmol/L)	8.5 ± 3.81	8.04 ± 3.88	0.630
HbA1c (%)	6.05 ± 0.88	6.01 ± 0.88	0.882
HOMA-beta^§^	65.87 (45.88–90.99)	69.26 (41.86–97.28)	0.754
HOMA-IR^§^	1.50 (1.18–2.01)	1.68 (1.24–2.68)	0.263
Adiponectin (μg/ml)^§^	5.35 (3.00–7.31)	4.64 (2.40–5.60)	0.089
FGF21 (pg/ml)^§^	382.35 (261.63–609.00)	350.32 (191.70–590.60)	0.441
CK18 M30 (U/L)^§^	115.69 (84.37–169.18)	181.68 (115.45–297.37)	0.004
CK18 M65ED (U/L)^§^	244.40 (139.30–387.43)	398.1 (191.75–867.65)	0.002

Data are means ± SD or median (interquartile range). ^§^Ln transformed before analysis.

Using univariate logistic regression analysis, the OR for ln M30 and ln M65ED in predicting suspected NASH were 3.521 (95%CI 1.409–8.798; P = 0.007) and 2.897 (95%CI 1.412–5.944; P = 0.004), respectively (Supplementary Table S4). Notably, M65ED was still an independent predictor of suspected NASH (OR: 3.250 [95%CI 1.196–8.826]; P = 0.021) in a multivariate model including age, SBP, AST and GGT. However, M30 was not an independent predictor (OR: 2.789 [95%CI 0.932–8.344]; P = 0.067) after adjustment for other related baseline parameters (Supplementary Table S4). These results suggested the elevation of CK18 M30 and M65ED levels in NAFLD patients was correlated with an ominous prognosis.

### Expression of FGF21, CK18 and CK18 M30 in human liver tissue examined by immunohistochemistry staining

To determine if FGF21, CK18 and CK18 M30 expression levels in liver were changed along with liver pathological manifestations and apoptosis marker, we examined the markers using immunohistochemistry staining in liver tissues from patients without NAFLD, patients with simple steatosis or with NASH. Representative images were shown in Supplementary Fig. S2. FGF21 expression were elevated in liver tissues from patients who were diagnosed as simple steatosis or NASH. CK18 was expressed in almost all hepatocytes, while CK18 fragment M30 and apoptosis marker cleaved-caspase3 were identified only in NASH patients but were rarely detected in patients with simple steatosis or patients without NAFLD.

### Predictive value of FGF21, M30 and M65ED levels in the onset of simple steatosis, remission and the development of suspected NASH

**Figure 2:**
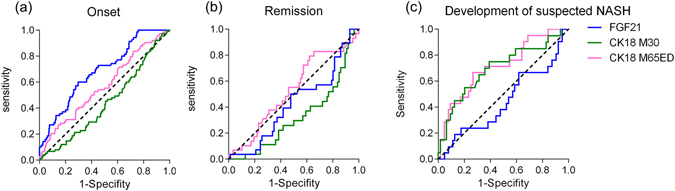
ROC curves for predicting the development and progression of NAFLD. (**a**) ROC curves of FGF21, M30 and M65ED for predicting the onset of simple steatosis. (**b**) ROC curves for predicting the remission of NAFLD during follow-up. (**c**) ROC curves for predicting suspected NASH in subjects who sustained NAFLD.

The receiver-operating characteristic (ROC) curves shown in Fig. 2a represented the predictive accuracy of the three biomarkers for the onset of simple steatosis. FGF21 [0.661 (0.589–0.773); P < 0.001] can predict the onset of simple steatosis while M30 [0.427 (0.353–0.502); P = 0.077] cannot, and M65ED showed an area under curve (AUC) of 0.581 (0.502–0.660); P = 0.049. Lower M30 level [0.675 (95%CI 0.563–0.788; P = 0.006)] was an independent predictor of NAFLD remission during follow-up while FGF21 [0.574 (95%CI 0.455–0.693; P = 0.243)] and M65ED [0.469 (95%CI 0.348–0.589; P = 0.614)] were not predictive of NAFLD remission (Fig. 2b). We also found the AUC for predicting suspected NASH in subjects who sustained NAFLD by M65ED and M30 were 0.714 (95%CI 0.586–0.843; P = 0.003) and 0.698 (95%CI 0.558–0.838; P = 0.008), respectively (Fig. 2c). However, FGF21 was not able to predict suspected NASH with an AUC of 0.444 (95%CI 0.297–0.592; P = 0.448).

### Potential positive relationship between baseline CK18 and liver stiffness evaluated by FibroScan after 8-year follow-up

**Figure 3:**
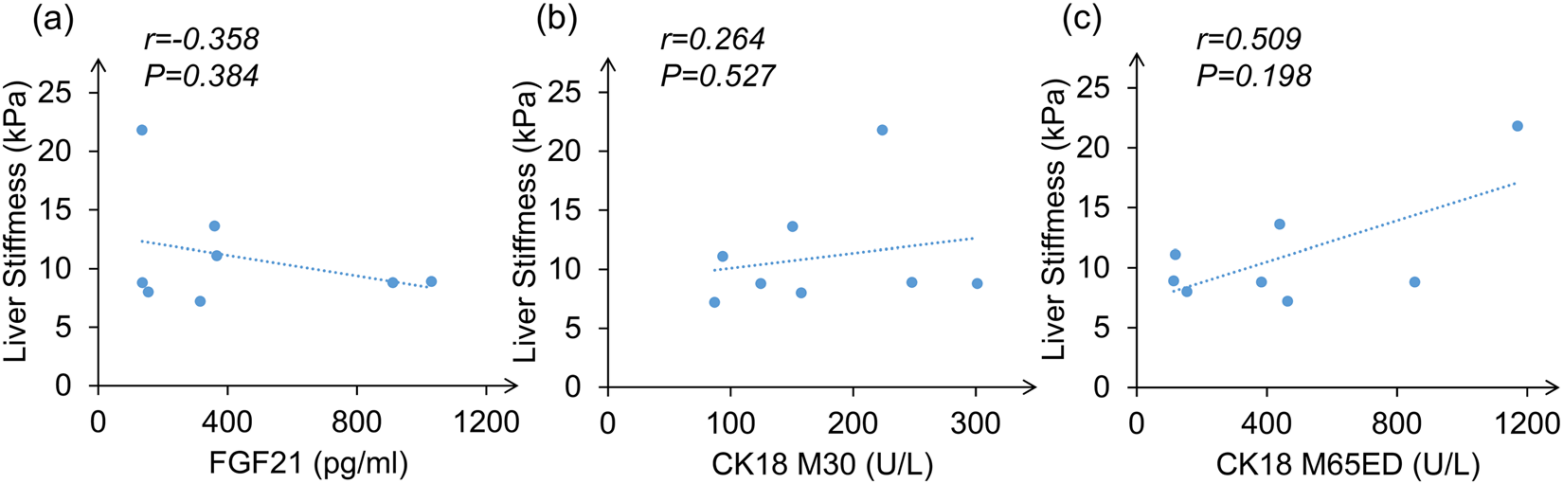
Relationship between baseline FGF21, CK18 M30 and CK18 M65ED with liver stiffness after 8-year follow-up. (**a**) Correlation of baseline FGF21 levels and liver stiffness evaluated by FibroScan after 8-year follow-up. (**b**) Correlation of baseline CK18 M30 levels and liver stiffness. (**c**) Correlation of baseline CK18 M65ED levels and liver stiffness.

To assess the relationship between levels of biomarkers at baseline and the development of liver fibrosis during follow-up, a subgroup from “suspected NASH” patients in 2011 were followed up for another 5 years. In 2016, 8 patients who had suspected NASH in 2011 received FibroScan test and results showed that they all had significant hepatic fibrosis (>7.0 kPa). The relationship between baseline levels of circulating biomarkers and liver stiffness after 8-year follow-up were displayed in Fig. 3. There is a non-significant trend of positive relationship between baseline CK18 and liver stiffness after 8-year follow-up. Baseline M65ED seemed to have a more obvious correlation with liver stiffness than M30. No similar relationship was found between FGF21 and liver stiffness.

## Discussion

We found for the first time that FGF21 and CK18, both are believed to be potential biomarkers for NAFLD, may play differential roles along the spectrum of NAFLD. FGF21 is more accurate for predicting the onset of simple steatosis, while CK18 including M30 and M65ED are better non-invasive biomarkers for predicting the prognosis of NAFLD patients.

Fat accumulation in NAFLD is due to increased delivery of free fatty acid (FFA) into the portal vein for conversion to TGs within the liver. It is well established that FGF21 was stimulated by FFA via PPARα activation^19^. Lipid infusion in humans induces an elevation in serum FGF21 levels, with a strong correlation between the changes in FGF21 and FFA levels^19^. Many animal studies have suggested FGF21 to exert protective impact on glucose and lipid metabolism through both endocrine and autocrine pathways, possibly as an attempt to maintain metabolic homeostasis^10, 11^. However, diet-induced obesity was proposed as an FGF21-resistant state, including attenuated FGF21 signaling response as assessed by extracellular mitogen-activated protein kinase 1 and 2 (ERK1/2) phosphorylation as well as an impaired induction of FGF21 target genes, including cFos and EGR1 in both liver and fat^20^. Recent study also suggested that adipose tissue inflammation in obesity might repress the expression of FGF21 co-receptor beta-klotho via the JNK1 pathway^21^. The increase of FGF21 was considered as a compensatory response to decreased FGF21 sensitivity in diet-induced obesity^20^.

In previous human study, we found a significantly positive association of both serum concentrations and liver mRNA expression of FGF21 with TG. FGF21 mRNA expression in liver tissues of Grade 1 steatosis (6–33%) was more than 4-fold higher than that in Grade 0 (0–5%)^12^. Our immunohistochemistry staining results showed that FGF21 expression was elevated in liver from patients with simple steatosis. Those findings suggested that FGF21 might be sensitive in detecting mild steatosis. Although various metabolic risk factors including baseline HOMA values, lower adiponectin levels and abnormal lipid profile were reported to be related with NAFLD^22–24^, we found FGF21 was an independent predictor of the onset of simple steatosis when baseline parameters were included in multiple models. M30 was reported to be more accurate in diagnosing NAFLD than FGF21 in a cross-sectional study^16^, our current prospective study demonstrated that neither baseline M30 nor M65ED level can independently predict the onset of simple steatosis. This was also supported by publication reported that FGF21 level was positively correlated with hepatic fat content in mild or moderate NAFLD patients, but decreases in severe NAFLD patients^25^. FGF21 level was also found to be increased in NAFLD but decreased in NASH patients^26^. In line with the above results, we found that baseline FGF21 was not able to predict the remission of NAFLD or suspected NASH at follow-up, suggesting FGF21 might not be a potential biomarker for the prognosis of NAFLD patients.

Unlike FGF21, CK18 is the major intermediate filament protein in the hepatocyte. Under physiological condition, the major function of CK18 is assumed to be a mechanical stress absorber and an integrating device for the entire cytoskeleton^27^. It is one of the most prominent substrates of caspases during apoptosis^28^. We found in immunohistochemistry staining that CK18 was expressed in almost all hepatocytes while CK18 fragment M30 was only identified in liver sample from NASH patients. CK18 fragment M30 is then released to circulation and can be detected using M30-based ELISA and served as apoptosis biomarker. M65ED-based ELISA detects the common epitope in both uncleaved and caspase-cleaved CK18^29, 30^. Although the significance of cytokeratin degradation during apoptosis is unclear, it is suggested that caspase cleavage of the cytokeratin proteins including CK18 is likely to facilitate the formation of apoptotic bodies and amplify the apoptotic signal^28^, as reflected by our immunohistochemistry staining showing that M30 and cleaved-caspase3 were elevated in liver from NASH patients but rarely detected in simple steatosis and normal liver tissue. Hepatocyte apoptosis is increased in patients with NASH than simple steatosis and correlates with disease severity^31^. Moreover, chronic elevated apoptosis of hepatocytes may directly stimulate fibrogenesis^31^.

In previous studies, M30 was found to be higher in NASH than “non-NASH NAFLD”^13, 16^. CK18 M30 level is also correlated with fibrosis in children with NAFLD^32^. Other studies also found in patients with chronic hepatitis C, M30 is associated with fibrosis instead of steatosis^33^. Subsequent studies showed a modest value of M30 in diagnosing NASH and fibrosis^15, 34^. Many previous studies confirmed that circulating levels of liver enzymes including ALT, AST and GGT were elevated in NAFLD patients^6^. However, according to our data, the levels of liver enzymes cannot predict the remission of NAFLD, suggesting a cross-sectional relationship between liver enzymes levels and NAFLD. When included in multiple regression model, only waist circumference and M30 were independent baseline predictors of the remission of NAFLD. The relationship between lower M30 level and NAFLD remission was also independent of lifestyle intervention during follow-up while baseline ALT levels cannot predict the remission of NAFLD, suggesting lower baseline M30 and milder central obesity are representative of a reversible status of the disease afterwards. Our result was in line with a previous immunohistochemistry study in human liver tissue using the M30 antibody, which showed that M30 antigen was identified in liver tissue from patients with biopsy-proved NASH. However, M30 was rarely detected in liver tissue of patients with simple steatosis^35^. From this perspective, it is possible that the dynamic change of M30 is useful for longitudinal evaluation of NAFLD patients and measuring response to intervention and therapy.

M65 and M65ED levels were previously reported to have diagnostic value for NASH and fibrosis in NAFLD patients^15, 36^. M65 was also found to be correlated with severe fibrosis in heavy alcohol drinkers^37^. M65ED can discriminate patients with different fibrosis stages^15^. Another study found that M65 can predict survival after acute liver failure^38^. M65 levels were also elevated in alcoholic liver fibrosis^37^. A more recent study also found that M65 levels were significantly correlated with overall survival of hepatocellular carcinoma patients^39^. These findings showed M65 or M65ED levels were correlated with multiple liver diseases which involved various forms of hepatocyte death. In our cohort, baseline M65ED was found to independently predict suspected NASH in subjects who sustained NAFLD. The predictive effect was not attenuated after adjustment for baseline clinical covariates. Moreover, there is a trend of positive correlation between baseline M65ED level and liver stiffness evaluated by FibroScan after 8-year follow-up although the relationship does not reach statistical significance. This result further suggested that M65ED might be a prospective biomarker for an unfavorable prognosis of NAFLD patients, including NASH, fibrosis and even cirrhosis.

Our study is the first prospective study to compare and clarify the different roles of FGF21, CK18 and CK18 fragment in early identification and monitoring of NAFLD patients in one cohort. Our results demonstrated that FGF21 is more accurate for predicting the onset of simple steatosis, while CK18 including M30 and M65ED are better biomarkers for monitoring the prognosis of NAFLD patients. FGF21, M30 and M65ED may play differential roles along the spectrum of NAFLD. Our study was limited by using ALT level instead of liver biopsy to define simple steatosis and suspected NASH. This study was not intended to validate diagnostic criteria for NAFLD but rather to explore the differential roles of FGF21 and CK18 in monitoring the different stages of NAFLD. Our results were also limited by the small sample size in the analysis of the relationship between baseline biomarker levels and liver stiffness at follow-up. Additional follow-up reassessment with liver biopsy will be valuable for validating the predictive effect of M30 and M65ED on NASH and liver fibrosis. Combination of CK18 with other potential NASH biomarkers including circulating oxidized fatty acids might be helpful for early detection and intervention for progressive subgroup in NAFLD patients.

## Methods

### Study subjects

This multi-stage stratified epidemiological study was designed to assess the prevalence of related diseases in a community in Shanghai^7^. A total of 808 Chinese origins (Han Chinese) aged from 20 to 79 years were enrolled from June to August 2008. All the subjects were invited for follow-up assessments in July to September 2011. A subgroup selected from “suspected NASH” patients in 2011 by random sampling were followed up for another 5 years. The subgroup received liver FibroScan® test (Echosens, Paris, France) to measure their liver stiffness in 2016. The demographic data were ascertained at each assessment. Detailed medical, drug and family histories, together with diet and physical exercise condition, were obtained using a standardized questionnaire. Subjects attended all assessments after an overnight fasting and underwent comprehensive physical examinations, routine biochemical analyses of blood, 75-g oral glucose tolerance test, hepatitis B surface antigen, hepatitis C virus antibody and B ultrasonography.

Subjects with following conditions were excluded from this study: acute or chronic virus hepatitis, drug-induced liver disease, biliary obstructive diseases, total parenteral nutrition, autoimmune hepatitis, Wilson’s disease, known hyperthyroidism or hypothyroidism, presence of cancer, current treatment with systemic corticosteroids and pregnancy. Current drinkers and ex-drinkers were excluded from the study. The study was approved by the human research ethics committee of the Shanghai Sixth People’s hospital, following the principles of the declaration of Helsinki. Written informed consent was obtained from all subjects.

### Human liver tissue

The liver tissues were previously collected from patients of benign focal hepatic lesions undergoing liver surgery at the Department of Liver Surgery (Zhongshan Hospital, Fudan University, Shanghai, China). Samples from patients complicated with hepatitis or chronic alcohol use were excluded. All tissue samples had been examined by a pathologist who was blinded to the study design. Simple steatosis is defined as the presence of hepatic steatosis with no evidence of hepatocellular injury in the form of ballooning of the hepatocytes^40^. NASH is defined as the presence of hepatic steatosis and inflammation with hepatocyte injury (ballooning) with or without fibrosis^40^. The study was approved by the local ethics committee, following the principles of the Declaration of Helsinki. Written informed consent was obtained from each patient.

### Clinical diagnosis for NAFLD

Guidelines for the diagnosis of NAFLD proposed by the Asia-Pacific Working Party were used^41^. NAFLD was clinically defined as manifestations of B ultrasonography respectively at baseline and at follow-up assessment, ruling out the habit of drinking and the history of specific diseases that could result in fatty liver. In B ultrasonography, hepatic steatosis was defined as a diffuse increase of fine echoes in the liver parenchyma compared with that in the kidney or spleen parenchyma based on standard criteria. Abdominal ultrasonography was performed by experienced radiologists who were blinded to clinical presentation and laboratory findings. In this study, subjects with NAFLD at baseline and diagnosed as non-NAFLD at follow-up were defined as remission. Simple steatosis was defined as NAFLD patients with ALT level <40U/L and suspected NASH was defined as NAFLD patients with ALT level ≥40U/L. Liver stiffness was measured using the FibroScan® medical device (Echosens, Paris, France). FibroScan® was performed by experienced physicians who were blinded to clinical findings. The procedure was based on at least ten validated measurements. The median value, expressed in kilopascals (kPa), was considered representative of the liver stiffness value. The liver stiffness value was considered reliable only if at least 10 successful acquisitions were obtained.

### Anthropometric and biochemical measurements

Waist circumference, body fat percentage, BMI, HbA_1c_, serum insulin and other biochemical indexes were measured as previously described^7^. Basal insulin secretion and insulin sensitivity were estimated by the HOMA^42^. Subjects who had regular exercise during follow-up were defined as subjects who had moderate-to-vigorous exercise for at least 4 days per week (at least 150 minutes per week). Low calorie diet was defined as intake reduction by 500–1000 kcal/day^43^.

Concentrations of FGF21 and adiponectin in serum were quantified using enzyme-linked immunosorbent assay kits as previously reported (Antibody and Immunoassay Services, the University of Hong Kong)^7^. The serum concentration of M30 was determined using M30-Apoptosense enzyme-linked immunosorbent assay kit (Peviva AB, Bromma, Sweden) according to the manufacturer’s instructions. M65ED was determined with the M65 EpiDeath enzyme-linked immunosorbent assay (Peviva AB, Bromma, Sweden). The M30 and M65ED concentrations were expressed as units per liter (U/L). The intra- and inter assay variations of M30 measurement were 2.0 and 8.5%, respectively. The intra- and inter assay variations of M65ED measurement were 3.4 and 9.0%, respectively. As reported in our previous publication, the intra- and inter assay variations of FGF21 were 4.4% and 9.2%, respectively^7^.

### Immunohistochemistry

Paraffin-embedded liver tissue was cut, deparaffinized, hydrated and antigen retrieval was performed using preheated citrate buffer. Immunohistochemistry was performed with rabbit polyclonal FGF21 antibody (Abcam, Cambridge, UK), mouse monoclonal M30 antibody (Peviva AB, Bromma, Sweden), mouse monoclonal CK18 antibody (GoodBio, Wuhan, China) and rabbit polyclonal cleaved-caspase3 antibody (GoodBio, Wuhan, China) for detection of the various markers. The samples were incubated with the primary antibody overnight at 4 °C. After washing with phosphate-buffered saline, the sections were incubated with HRP-secondary antibody (DAKO Corporation, Carpinteria, CA) for 50 minutes at room temperature. After washing with phosphate-buffered saline, the samples were stained with 3,3-diaminobenzidine (DAKO Corporation, Carpinteria, CA) for 2 to 5 minutes, washed in phosphate-buffered saline, counterstained with hematoxylin for 2 to 3 minutes, and dehydrated by transferring them through increasing ethanol solutions (75%, 85%, 100%, 100% ethanol). Following dehydration, the slices were soaked in a xylene bath at room temperature for 5 minutes, mounted, and examined.

### Statistical analysis

All statistical analyses were performed with SPSS Version 20.0 (SPSS Inc., Chicago, IL). Normally distributed data were expressed as mean ± SD. Data that were not normally distributed, as determined by using the Shapiro-Wilk test, were ln transformed before analysis and expressed as median with interquartile range. Student’s unpaired t test was used for comparison between two groups. Chi-square tests were used to compare difference in proportions of categorical variables between groups. Pearson’s and partial correlation were used to examine the association among parameters. Univariate logistic regression was performed to analyze the value of a single baseline parameter to predict the onset or progression of NAFLD. To identify independent predictors, baseline variables that were significantly different between two groups and were biologically likely to be related with NAFLD or NASH were analyzed using multiple logistic regressions. OR per standard deviation was used to show the relative strength of the relationship. The predictive accuracy of the three biomarkers were evaluated respectively using ROC-AUC with 95% CI. Two-sided P values < 0.05 were considered significant.

## Electronic supplementary material

Supplementary information

## References

[1] Neuschwander-Tetri BA, Caldwell SH (2003). Nonalcoholic steatohepatitis: summary of an AASLD Single Topic Conference. Hepatology.

[2] Kleiner DE (2005). Design and validation of a histological scoring system for nonalcoholic fatty liver disease. Hepatology.

[3] Yilmaz Y (2012). Review article: is non-alcoholic fatty liver disease a spectrum, or are steatosis and non-alcoholic steatohepatitis distinct conditions?. Alimentary pharmacology & therapeutics.

[4] Ratziu V (2005). Sampling variability of liver biopsy in nonalcoholic fatty liver disease. Gastroenterology.

[5] Khov N, Sharma A, Riley TR (2014). Bedside ultrasound in the diagnosis of nonalcoholic fatty liver disease. World journal of gastroenterology.

[6] Machado MV, Cortez-Pinto H (2013). Non-invasive diagnosis of non-alcoholic fatty liver disease. A critical appraisal. Journal of hepatology.

[7] Li H (2013). High serum level of fibroblast growth factor 21 is an independent predictor of non-alcoholic fatty liver disease: a 3-year prospective study in China. Journal of hepatology.

[8] Li H, Zhang J, Jia W (2013). Fibroblast growth factor 21: a novel metabolic regulator from pharmacology to physiology. Frontiers of medicine.

[9] Dutchak PA (2012). Fibroblast growth factor-21 regulates PPARgamma activity and the antidiabetic actions of thiazolidinediones. Cell.

[10] Fisher FM (2014). Fibroblast growth factor 21 limits lipotoxicity by promoting hepatic fatty acid activation in mice on methionine and choline-deficient diets. Gastroenterology.

[11] Woo YC, Xu A, Wang Y, Lam KS (2013). Fibroblast growth factor 21 as an emerging metabolic regulator: clinical perspectives. Clinical endocrinology.

[12] Li H (2010). Fibroblast growth factor 21 levels are increased in nonalcoholic fatty liver disease patients and are correlated with hepatic triglyceride. Journal of hepatology.

[13] Feldstein AE (2009). Cytokeratin-18 fragment levels as noninvasive biomarkers for nonalcoholic steatohepatitis: a multicenter validation study. Hepatology.

[14] Feldstein AE (2003). Hepatocyte apoptosis and fas expression are prominent features of human nonalcoholic steatohepatitis. Gastroenterology.

[15] Joka D (2012). Prospective biopsy-controlled evaluation of cell death biomarkers for prediction of liver fibrosis and nonalcoholic steatohepatitis. Hepatology.

[16] Shen J (2012). Non-invasive diagnosis of non-alcoholic steatohepatitis by combined serum biomarkers. Journal of hepatology.

[17] Siest G (1975). Aspartate aminotransferase and alanine aminotransferase activities in plasma: statistical distributions, individual variations, and reference values. Clinical chemistry.

[18] Sherman KE (1991). Alanine aminotransferase in clinical practice. A review. Arch Intern Med.

[19] Mai K (2009). Free fatty acids link metabolism and regulation of the insulin-sensitizing fibroblast growth factor-21. Diabetes.

[20] Fisher FM (2010). Obesity is a fibroblast growth factor 21 (FGF21)-resistant state. Diabetes.

[21] Diaz-Delfin J (2012). TNF-alpha represses beta-Klotho expression and impairs FGF21 action in adipose cells: involvement of JNK1 in the FGF21 pathway. Endocrinology.

[22] Zelber-Sagi S (2012). Predictors for incidence and remission of NAFLD in the general population during a seven-year prospective follow-up. Journal of hepatology.

[23] Kamada Y, Takehara T, Hayashi N (2008). Adipocytokines and liver disease. J Gastroenterol.

[24] Hamaguchi M (2005). The metabolic syndrome as a predictor of nonalcoholic fatty liver disease. Ann Intern Med.

[25] Yan H (2011). Circulating fibroblast growth factor 21 levels are closely associated with hepatic fat content: a cross-sectional study. PloS one.

[26] Dushay J (2010). Increased fibroblast growth factor 21 in obesity and nonalcoholic fatty liver disease. Gastroenterology.

[27] Herrmann H, Bar H, Kreplak L, Strelkov SV, Aebi U (2007). Intermediate filaments: from cell architecture to nanomechanics. Nature reviews. Molecular cell biology.

[28] Caulin C, Salvesen GS, Oshima RG (1997). Caspase cleavage of keratin 18 and reorganization of intermediate filaments during epithelial cell apoptosis. J Cell Biol.

[29] Kramer G (2004). Differentiation between cell death modes using measurements of different soluble forms of extracellular cytokeratin 18. Cancer research.

[30] Luft T (2007). Serum cytokeratin-18 fragments as quantitative markers of epithelial apoptosis in liver and intestinal graft-versus-host disease. Blood.

[31] Chakraborty JB, Oakley F, Walsh MJ (2012). Mechanisms and biomarkers of apoptosis in liver disease and fibrosis. Int J Hepatol.

[32] Mandelia C (2016). Plasma Cytokeratin-18 Level As a Novel Biomarker for Liver Fibrosis in Children With Nonalcoholic Fatty Liver Disease. Journal of pediatric gastroenterology and nutrition.

[33] Jazwinski AB (2012). Elevated serum CK18 levels in chronic hepatitis C patients are associated with advanced fibrosis but not steatosis. Journal of viral hepatitis.

[34] Cusi K (2014). Limited value of plasma cytokeratin-18 as a biomarker for NASH and fibrosis in patients with non-alcoholic fatty liver disease. Journal of hepatology.

[35] Wieckowska A (2006). *In vivo* assessment of liver cell apoptosis as a novel biomarker of disease severity in nonalcoholic fatty liver disease. Hepatology.

[36] Shen J (2012). Assessment of non-alcoholic fatty liver disease using serum total cell death and apoptosis markers. Alimentary pharmacology & therapeutics.

[37] Lavallard VJ (2011). Serum markers of hepatocyte death and apoptosis are non invasive biomarkers of severe fibrosis in patients with alcoholic liver disease. PloS one.

[38] Bechmann LP (2010). Cytokeratin 18-based modification of the MELD score improves prediction of spontaneous survival after acute liver injury. Journal of hepatology.

[39] Waidmann O (2013). Diagnostic and prognostic significance of cell death and macrophage activation markers in patients with hepatocellular carcinoma. Journal of hepatology.

[40] Chalasani N (2012). The diagnosis and management of non-alcoholic fatty liver disease: practice Guideline by the American Association for the Study of Liver Diseases, American College of Gastroenterology, and the American Gastroenterological Association. Hepatology.

[41] Chitturi S (2007). Non-alcoholic fatty liver disease in the Asia-Pacific region: definitions and overview of proposed guidelines. Journal of gastroenterology and hepatology.

[42] Matthews DR (1985). Homeostasis model assessment: insulin resistance and beta-cell function from fasting plasma glucose and insulin concentrations in man. Diabetologia.

[43] Jian-gao F, Chinese Liver Disease A (2010). Guidelines for management of nonalcoholic fatty liver disease: an updated and revised edition. Zhonghua Gan Zang Bing Za Zhi.

