# Supplementary material for: Complementary Role of Fibroblast Growth Factor 21 and Cytokeratin 18 in Monitoring the Different Stages of Nonalcoholic Fatty Liver Disease
Source: Sci Rep. 2017 Jul 11;7:5095. doi: 10.1038/s41598-017-05257-5 (PMC5506050; doi:10.1038/s41598-017-05257-5)
Supplement: Supplementary file 1 — Supplementary information [file 41598_2017_5257_MOESM1_ESM.pdf]

Complementary Role of Fibroblast Growth Factor 21 and Cytokeratin 18 in Monitoring the Different Stages of Nonalcoholic Fatty Liver Disease

Guangyu Wu[†], Huating Li[†], Qichen Fang, Jing Zhang, Mingliang Zhang, Lei Zhang, Liang Wu, Xuhong Hou, Junxi Lu, Yuqian Bao, and Weiping Jia[*]

Supplementary Information

Supplementary Figure S1 Flow chart of the study.

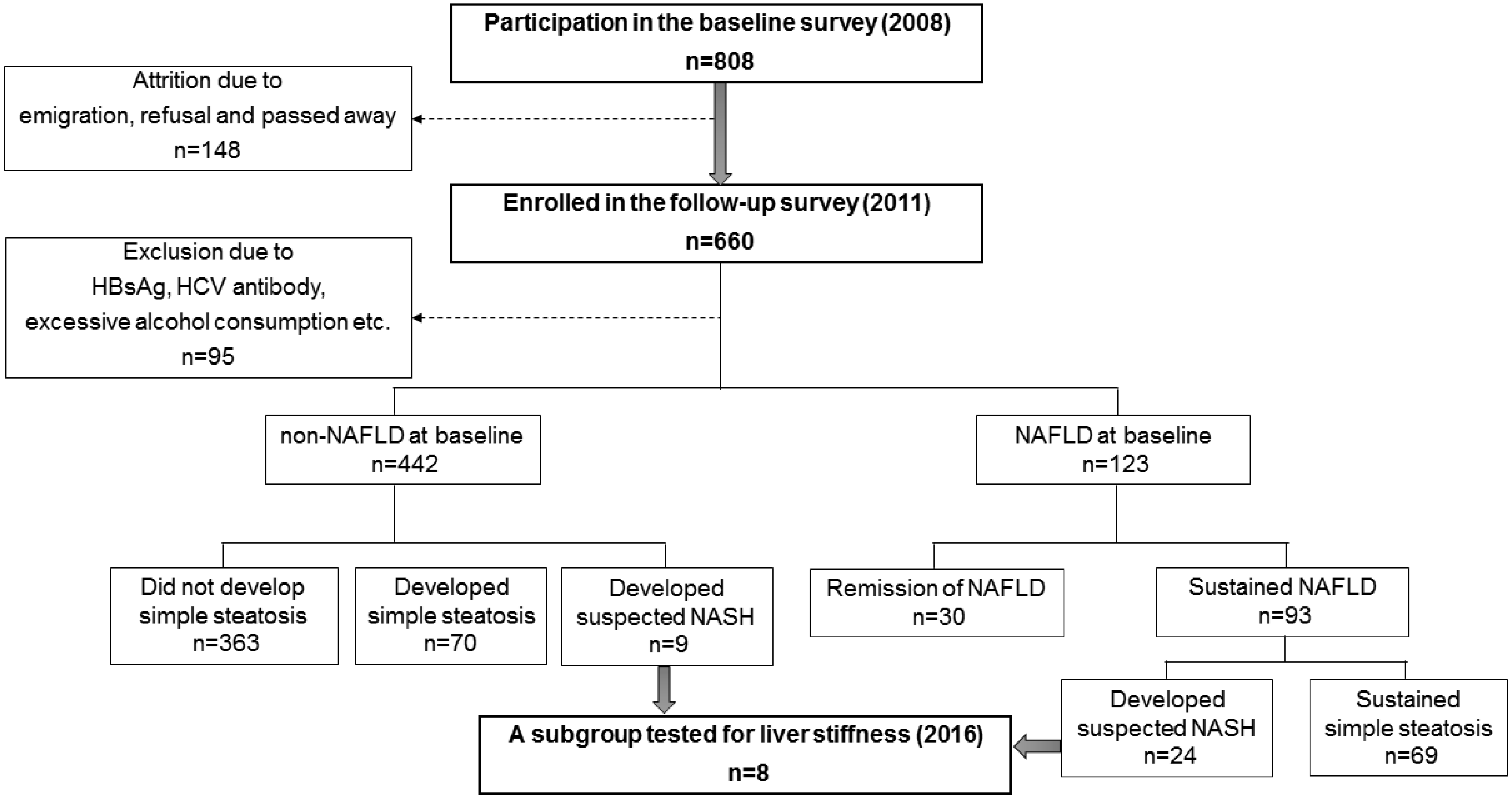

Supplementary Figure S2 Expression of FGF21, CK18, CK18 M30 and apoptosis marker in human liver tissue examined by immunohistochemistry staining.

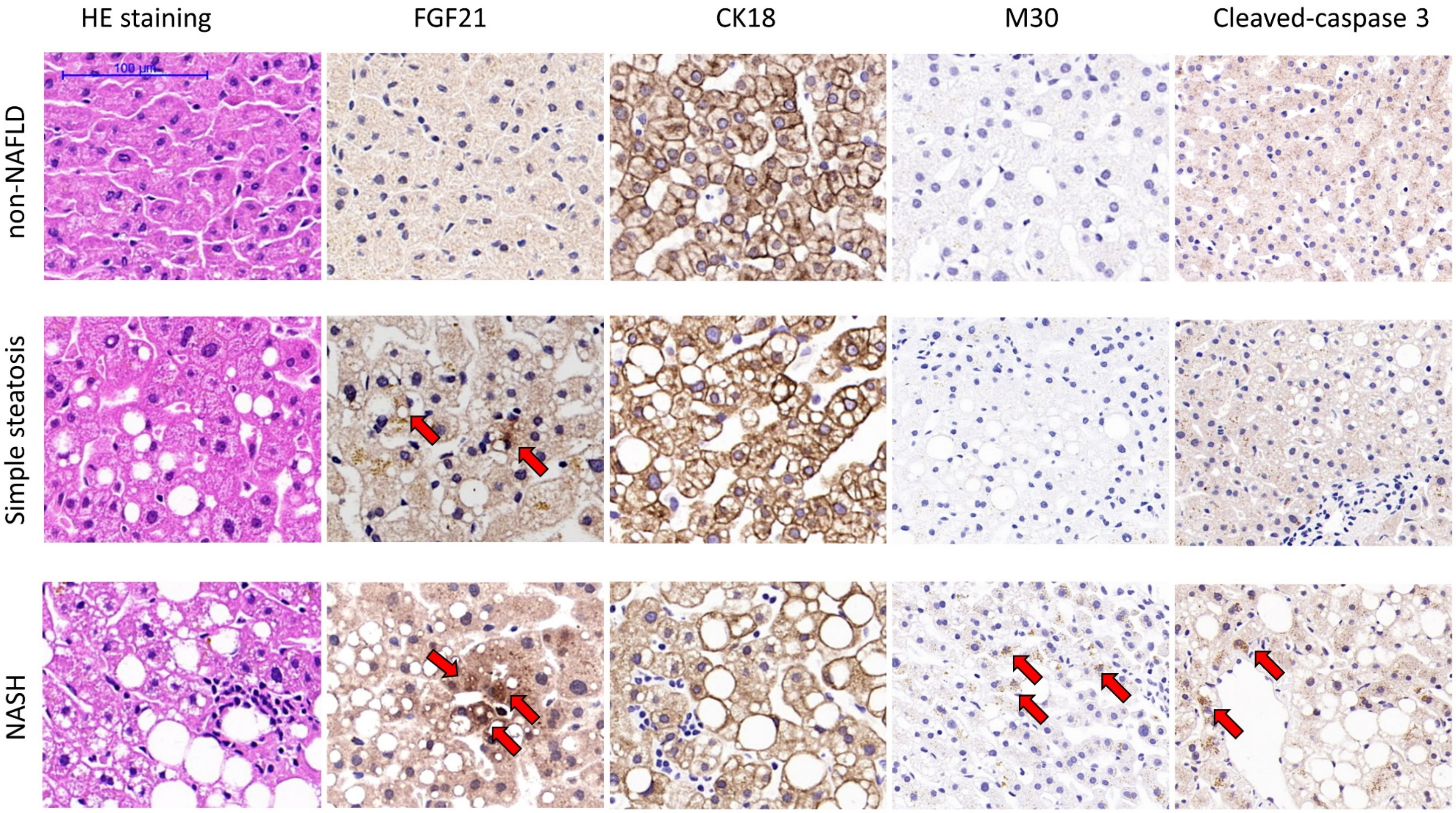

Supplementary Table S1 Pearson and partial correlations between baseline FGF21 and cell death biomarkers and clinical parameters in total cohort.

Baseline variables	FGF21		CK18 M30		CK18 M65ED		Adjusted for age and BMI					
							FGF21		CK18 M30		CK18 M65ED	
	r	*p*	r	*p*	r	*p*	r	*p*	r	*p*	r	*p*
Age (years)	0.303	<0.001	-0.049	0.261	0.288	<0.001						
BMI (kg/m^2)	0.324	<0.001	0.094	0.032	0.208	<0.001						
Waist circumference (cm)	0.418	<0.001	0.090	0.039	0.296	<0.001	0.244	<0.001	0.037	0.395	0.186	<0.001
SBP (mmHg)	0.301	<0.001	0.023	0.603	0.303	<0.001	0.105	0.019	0.016	0.711	0.158	<0.001
DBP (mmHg)	0.313	<0.001	0.050	0.257	0.302	<0.001	0.146	0.001	0.034	0.440	0.182	<0.001
ALT (IU/L)[§]	0.220	<0.001	0.239	<0.001	0.546	<0.001	0.097	0.029	0.227	<0.001	0.516	<0.001
AST (IU/L)[§]	0.180	<0.001	0.176	<0.001	0.543	<0.001	0.037	0.411	0.188	<0.001	0.480	<0.001
GGT (IU/L)[§]	0.327	<0.001	0.154	<0.001	0.409	<0.001	0.207	<0.001	0.141	0.001	0.343	<0.001
TC (mmol/L)	0.283	<0.001	0.095	0.029	0.257	<0.001	0.184	<0.001	0.106	0.015	0.168	<0.001
TG (mmol/L)[§]	0.450	<0.001	0.051	0.242	0.348	<0.001	0.342	<0.001	0.023	0.599	0.268	<0.001
HDL-C (mmol/L)	-0.257	<0.001	-0.003	0.937	-0.148	0.001	-0.181	<0.001	0.030	0.488	-0.095	0.029
LDL-C (mmol/L)	0.139	0.002	0.107	0.014	0.132	0.002	0.060	0.182	0.106	0.016	0.068	0.122
FPG (mmol/L)	0.049	0.267	0.078	0.073	0.158	<0.001	-0.013	0.768	0.069	0.113	0.122	0.005
2hPG (mmol/L)	0.157	<0.001	0.091	0.037	0.238	<0.001	0.044	0.326	0.092	0.035	0.156	<0.001
HbA1c (%)	0.096	0.031	0.070	0.112	0.237	<0.001	-0.020	0.655	0.075	0.087	0.156	<0.001
Adiponectin (μg/ml)[§]	-0.194	<0.001	-0.109	0.015	-0.146	0.001	-0.166	<0.001	-0.064	0.153	-0.157	<0.001

[§]Ln transformed before analysis.

Supplementary Table S2 Baseline clinical parameters of 70 subjects who developed simple steatosis and 363 subjects who did not during 3-year follow-up.

Baseline variables	NAFLD state at 3 years		*P* value
	Did not develop simple steatosis (n=363)	Developed simple steatosis (n=70)	
M/F	131/232	24/46	0.684
Age (years)	44.50 ± 12.86	51.72 ± 13.32	<0.001
BMI (kg/m^2)	22.09 ± 2.72	24.80 ± 2.62	<0.001
Waist circumference (cm)	72.03 ± 7.62	79.41 ± 6.79	<0.001
Fat percentage (%)	24.81 ± 6.36	30.16 ± 6.90	<0.001
SBP (mmHg)	121.13 ± 16.16	127.79 ± 13.80	0.002
DBP (mmHg)	79.41 ± 9.47	84.76 ± 8.30	<0.001
ALT (IU/L)[§]	14.00 (11.00-18.00)	15.00 (12.00-20.00)	0.013
AST (IU/L)[§]	19.00 (16.00-22.00)	21.00 (17.00-23.50)	0.032
GGT (IU/L)[§]	16.00 (13.00-22.00)	19.00 (15.00-24.50)	0.033
TC (mmol/L)	4.58 ± 1.01	4.99 ± 0.96	0.002
TG (mmol/L)[§]	1.03 (0.74-1.38)	1.62 (1.05-1.94)	<0.001
HDL-C (mmol/L)	1.41 ± 0.30	1.30 ± 0.21	0.005
LDL-C (mmol/L)	2.81 ± 0.85	3.08 ± 0.79	0.015
FPG (mmol/L)	5.16 ± 1.05	5.15 ± 0.78	0.948
2hPG (mmol/L)	5.99 ± 2.16	6.98 ± 2.72	0.001
HbA1c (%)	5.61 ± 0.81	5.72 ± 0.50	0.284
HOMA-%B[§]	67.58 (46.25-96.50)	78.49 (55.02-118.78)	0.022
HOMA-IR[§]	1.15 (0.79-1.56)	1.42 (1.07-1.81)	0.013
Adiponectin (μg/ml)[§]	9.00 (6.40-13.57)	7.76 (5.29-11.64)	0.033
FGF21 (pg/ml)[§]	199.1 (123.56-322.8)	309.79 (169.11-506.43)	<0.001
CK18 M30 (U/L)[§]	87.12 (59.58-137.43)	77.96 (56.60-112.22)	0.240
CK18 M65ED (U/L)[§]	147.2 (98.90-234.90)	172.65 (112.98-301.05)	0.049

Data are means ± SD or median (interquartile range). [§]Ln transformed before analysis.

Supplementary Table S3 Baseline parameters predictive of NAFLD remission at 3 years in patients who had NAFLD at baseline, examined using logistic regression.

Baseline variables	Model 1	Model 2	Model 3	Model 4	Model 5
BMI				0.980 (0.746-1.286)	
Waist circumference				0.899 (0.809-0.999)*	
LDL-C				0.525 (0.269-1.025)	
FPG				0.819 (0.549-1.223)	
Exercise					2.845 (1.097-7.380)*
Diet control					0.624 (0.208-1.871)
FGF21	0.673 (0.341-1.331)				
CK18 M30		0.346 (0.147-0.814)*		0.364 (0.141-0.936)*	0.356 (0.147-0.859)*
CK18 M65ED			1.099 (0.649-1.862)		

Data are odds ratio (OR) (95%CI).
Model 1. Included only baseline FGF21 level.
Model 2. Included only baseline CK18 M30 level.
Model 3. Included only baseline CK18 M65ED level.
Model 4. Included BMI, waist circumference, LDL-C, FPG and CK18 M30.
Model 5. Included exercise, diet control and CK18 M30.
* P<0.05.

Supplementary Table S4 Baseline parameters predictive of suspected NASH at 3 years in patients who had NAFLD at baseline and sustained NAFLD during follow-up, examined using logistic regression.

Baseline variables	Model 1	Model 2	Model 3	Model 4	Model 5	Model 6
Age				0.935 (0.880-0.994)*	0.915 (0.859-0.975)*	
SBP				0.949 (0.900-1.002)	0.954 (0.904-1.006)	
AST				1.946 (0.307-12.345)	1.160 (0.139-9.682)	
GGT				1.702 (0.564-5.139)	1.771 (0.563-5.566)	
Exercise						0.663 (0.163-2.695)
Diet control						1.739 (0.541-5.594)
FGF21	0.738 (0.342-1.589)					
CK18 M30		3.521 (1.409-8.798)*		2.789 (0.932-8.344)		
CK18 M65ED			2.897 (1.412-5.944)*		3.250 (1.196-8.826)*	2.920 (1.398-6.100)*

Data are odds ratio (OR) (95%CI).
Model 1. Included only baseline FGF21 level.
Model 2. Included only baseline CK18 M30 level.
Model 3. Included only baseline CK18 M65ED level.
Model 4. Included age, SBP, AST, GGT and CK18 M30.
Model 5. Included age, SBP, AST, GGT and CK18 M65ED.
Model 6. Included exercise, diet control and CK18 M65ED.
* $P<0.05$.
